# Editorial: Cervical cancer control in Latin America and the Caribbean

**DOI:** 10.3389/fonc.2023.1226915

**Published:** 2024-05-08

**Authors:** Angélica Nogueira-Rodrigues, Lucely Cetina Perez, Mauricio Maza

**Affiliations:** ^1^ Department of General Medicine, Federal University of Minas Gerais, Belo Horizonte, Brazil; ^2^ Department of Clinical Research, Instituto Nacional de Cancerologia, National Cancer Institute, Mexico City, Mexico; ^3^ Pan American Health Organization, Washington, DC, United States

**Keywords:** cervical cancer 1, Latin America, epidemiology, prevention, screening, treatment, elimination

Worldwide, more than half a million women are diagnosed with cervical cancer (CC) annually, and over 300,000 die from the disease. Low-and middle-income countries (LMICs) account for around 85%, almost 10% of them in Latin America (LATAM) and the Caribbean, where mortality rates are almost five times higher than in high- income countries (HICs) (1, 2).

The long natural history of HPV-carcinogenesis provides a window of opportunity for secondary prevention with screening tests, which identify women infected with HPV or with cytologic abnormalities indicative of precancerous lesions. These lesions can be successfully controlled with early treatment (3). The existence of a primary infectious etiologic agent allows for primary prevention with prophylactic HPV vaccines capable of reducing the incidence of causative infections. Thus, cervical cancer is considered a preventable and treatable disease, despite the fact it continues to be the third highest cause of cancer in women in the region (3).

In May 2018, the World Health Organization (WHO) made a call to action for the global elimination of the disease as a public health problem. Elimination would occur when incidence rates scale down to less than 4 cases/100,000 women and would be possible through a strategy comprising three goals to be achieved by 2030: 90% HPV vaccination coverage of girls by 15 years of age, 70% screening coverage with high-performance tests of women by ages 35 and 45, and adequate management and treatment of 90% of precancerous lesions and invasive cancers (4). According to the WHO’s predictions, in LMICs and in most countries of Latin America, CC elimination is possible in the long term but will depend heavily on achieving the target for vaccination coverage (5, 6).

The main objectives of this Research Topic, comprised of nine articles, are to outline the most recent strategies to control CC in the sovereign states of LATAM, to present obstacles to disease control in the region, and to discuss ideas to overcome them. 

Starting from vaccination, the advent of HPV prophylactic vaccination offers a potential large step towards control of CC and other HPV-related cancers. Based on the high incidence of HPV-related cancers, the strong carcinogenic potential of certain HPV strains, and numerous trials proving the high efficacy of vaccines, immunization is considered one of the most important tools to alter the incidence of HPV-associated cancers in LMICs globally (7, 8). However, this Research Topic brings data to alert that HPV vaccine uptake in LATAM has been lower than expected. In the article from Nogueira-Rodrigues et al., a significant decline in its adhesion is reported, and several reasons are probably involved including limited knowledge of HPV and the HPV vaccine, misguided safety concerns, high cost, cultural barriers, and the COVID-19 pandemic. The authors present strategies to overcome the main barriers, such as adopting the one- dose schedule, delivering the vaccine to both health centers and schools, and advising health professionals to formally prescribe the vaccine.

Switching gears to screening strategies, in high-income countries, following the introduction of and adherence to Papanicolaou’s smear test in the 1940s, CC incidence declined by more than 60%, confirming this test as the most effective cancer screening tool in the history of medicine. However, the PAP smear has achieved limited success in LMICs due to several reasons, mainly lack of organized screening programs within weak health systems, technique limitations, low population coverage and not sufficiently reaching the high-risk population group, poor quality control, and insufficient monitoring (7). Furthermore, this dismal scenario has been significantly impacted by the COVID-19 pandemic with further declines at all levels of CC prevention and increasing inequalities, as reported by Cruz-Valdez et al.

In concordance with the WHO’s call for best practices to eliminate CC (4), the feasibility of self-collection of samples for high-risk HPV is currently being tested in several countries across the LATAM region, and a systematic review of the topic is presented in the Research Topic by Dartibale et al. HPV self-sampling is a promising strategy to overcome barriers to CC screening in areas with well-established screening programs, but may also reach those without organized screening and special populations such as indigenous, rural, and transgender women. Strategies to develop a concerted effort at local, regional, and national levels to support capacity building in reporting, monitoring, and surveilling, as well as strategies to comprehend and overcome cultural barriers for self-screening acceptance is shared by Mitchell et al., McFarlane et al., and Urrutia and Padilla.

Regarding treatment challenges, most CC patients in the region are diagnosed with locally advanced disease (9) and, since the late 1990s when a spate of US studies reported the benefit of cisplatin-based chemoradiation for CC, there has been a dearth of clinical advances in this setting and the cure rates of locally advanced disease have reached a plateau (10–13). Furthermore, efforts to increase disease control with additional chemotherapy have not been clearly positive so far (13–15). A systematic review and meta-analysis on concurrent chemoradiotherapy followed by adjuvant chemotherapy is presented in the Research Topic by Liu et al. Arango-Bravo et al. highlight a shortage in several aspects of CC treatment, including oncologists, chemotherapy units, and radiotherapy facilities, and that Mexico is a upper middle-income country country. To conclude, Maluf et al. make recommendations for the prevention, screening, diagnosis, staging, and management of CC in areas with limited resources based on an International Gynecological Cancer Society (IGCS) consensus meeting, defending that the development of guidelines by health care providers from LMI regions is more reflective of the reality on the ground.

Cervical cancer continues to be a public health challenge in LATAM and immediate coordinated efforts are urgently needed to best use the existing tools to control the disease. Given that HPV-associated tumors arise years, if not decades, after initial infection and that existing vaccines have no therapeutic efficacy on pre-existing CC (7), further delays to implement high- coverage HPV vaccination programs coupled with improvements in screening strategies will only mean continued loss of life from a preventable disease and undue financial burden on already constrained health systems.

## Author contributions

All authors contributed to conceptualization, data curation, formal analysis, validation, visualization; writing – original draft; writing – review & editing. All authors contributed to the article and approved the submitted version.
